# Supplementary material for: Assisted migration and the rare endemic plant species: the case of two endangered Mexican spruces
Source: PeerJ. 2022 Aug 3;10:e13812. doi: 10.7717/peerj.13812 (PMC9356587; doi:10.7717/peerj.13812)
Supplement: Supplemental Information 3 — Model fit metrics included the area under the receiver operator curve (AUC), the overall accuracy (OA), Matthews correlation coefficient (MCC), true skill statistic (TSS), Cohen’s kappa, sensitivity, specificity and probability of presence (PoP). [file peerj-10-13812-s003.docx]

| Species | Presences | Absences | Total | Prevalence | AUC | OA | MCC | TSS | Kappa | Sensitivity | Specificity | PoP_threshold_ |
| --- | --- | --- | --- | --- | --- | --- | --- | --- | --- | --- | --- | --- |
| Model for Mexico (30-arc second resolution) | | | | | | | | | | | | |
| *Picea martinezii* | 35 | 32,293 | 32,328 | 0.00108 | 0.986 | 0.999 | 0.971 | 0.971 | 0.971 | 0.971 | 1.000 | 0.73 |
| *Picea mexicana* | 38 | 32,571 | 32,609 | 0.00117 | 1.000 | 0.999 | 0.919 | 0.895 | 0.918 | 0.895 | 1.000 | 0.83 |
| World model (30-arc second resolution) | | | | | | | | | | | | |
| *Picea martinezii* | 46 | 22,004 | 22,050 | 0.00209 | 1.000 | 0.999 | 0.956 | 0.957 | 0.956 | 0.957 | 1.000 | 0.73 |
| *Picea mexicana* | 50 | 32,571 | 32,621 | 0.00154 | 1.000 | 0.999 | 0.904 | 0.922 | 0.904 | 0.922 | 1.000 | 0.83 |
