# Supplementary material for: Assisted migration and the rare endemic plant species: the case of two endangered Mexican spruces
Source: PeerJ. 2022 Aug 3;10:e13812. doi: 10.7717/peerj.13812 (PMC9356587; doi:10.7717/peerj.13812)
Supplement: Supplemental Information 4 [file peerj-10-13812-s004.docx]

| Class | Variable | Description | Source | *Picea martinezii* | | | |  | *Picea mexicana* | | | |
| --- | --- | --- | --- | --- | --- | --- | --- | --- | --- | --- | --- | --- |
|  |  |  |  | **Mean** | **SD** | **Min** | **Max** |  | **Mean** | **SD** | **Min** | **Max** |
| Climate | Bio_1 | Annual mean temperature (ºC) | WorldClim | 13.50 | 1.90 | 10.60 | 15.50 |  | 9.30 | 0.70 | 7.90 | 10.30 |
|  | Bio_2 | Mean diurnal range (mean of monthly (max temp - min temp)) (ºC) | WorldClim | 14.60 | 0.60 | 13.60 | 15.20 |  | 14.00 | 1.00 | 13.10 | 15.80 |
|  | Bio_3 | Isothermality (BIO2/BIO7) (* 100) (ºC) | WorldClim | 6.60 | 0.00 | 6.60 | 6.70 |  | 6.40 | 0.20 | 6.10 | 6.60 |
|  | Bio_4 | Temperature seasonality (standard deviation *100) (ºC) | WorldClim | 22.90 | 2.10 | 20.20 | 25.10 |  | 24.70 | 5.20 | 20.80 | 33.62 |
|  | Bio_5 | Max temperature of warmest month (ºC) | WorldClim | 24.10 | 2.10 | 21.10 | 26.40 |  | 20.00 | 1.40 | 17.90 | 22.70 |
|  | Bio_6 | Min temperature of coldest month (ºC) | WorldClim | 2.30 | 1.20 | 0.30 | 3.50 |  | -1.60 | 1.30 | -4.00 | -0.20 |
|  | Bio_7 | Temperature annual range (BIO5-BIO6) (ºC) | WorldClim | 21.90 | 1.00 | 20.50 | 22.90 |  | 21.70 | 2.30 | 19.90 | 25.60 |
|  | Bio_8 | Mean temperature of wettest quarter (ºC) | WorldClim | 14.90 | 1.70 | 12.00 | 16.60 |  | 11.50 | 1.30 | 9.50 | 13.90 |
|  | Bio_9 | Mean temperature of driest quarter (ºC) | WorldClim | 10.90 | 1.00 | 9.10 | 11.90 |  | 8.30 | 0.90 | 6.70 | 10.00 |
|  | Bio_10 | Mean temperature of warmest quarter (ºC) | WorldClim | 16.10 | 2.10 | 12.90 | 18.30 |  | 12.10 | 1.10 | 10.20 | 14.30 |
|  | Bio_11 | Mean temperature of coldest quarter (ºC) | WorldClim | 10.20 | 1.60 | 7.60 | 11.90 |  | 5.80 | 0.80 | 4.70 | 6.90 |
|  | Bio_12 | Annual precipitation (mm) | WorldClim | 787.90 | 67.90 | 654.00 | 855.00 |  | 858.40 | 209.00 | 686.00 | 1211.00 |
|  | Bio_13 | Precipitation of wettest month (mm) | WorldClim | 164.50 | 28.90 | 123.00 | 199.00 |  | 161.70 | 66.00 | 112.00 | 273.00 |
|  | Bio_14 | Precipitation of driest month (mm) | WorldClim | 19.10 | 2.10 | 17.00 | 22.00 |  | 23.80 | 1.60 | 22.00 | 27.00 |
|  | Bio_15 | Precipitation seasonality (coefficient of variation) | WorldClim | 68.50 | 7.50 | 59.00 | 77.00 |  | 62.40 | 13.20 | 54.00 | 85.00 |
|  | Bio_16 | Precipitation of wettest quarter (mm) | WorldClim | 359.90 | 41.00 | 292.00 | 409.00 |  | 426.00 | 173.20 | 301.00 | 717.00 |
|  | Bio_17 | Precipitation of driest quarter (mm) | WorldClim | 70.30 | 6.60 | 64.00 | 82.00 |  | 81.20 | 4.50 | 74.00 | 90.00 |
|  | Bio_18 | Precipitation of warmest quarter (mm) | WorldClim | 281.60 | 27.20 | 224.00 | 303.00 |  | 354.60 | 165.60 | 240.00 | 632.00 |
|  | Bio_19 | Precipitation of coldest quarter (mm) | WorldClim | 72.00 | 8.40 | 64.00 | 87.00 |  | 112.20 | 38.70 | 83.00 | 186.00 |
| Soil | DB | Absolute deep to bed rock (cm) | SoilGrids250m | 316.50 | 97.20 | 194.00 | 427.00 |  | 513.40 | 275.10 | 105.00 | 1128.00 |
|  | DB200 | Depth to bedrock (R horizon) up to 200 cm (cm) | SoilGrids250m | 156.30 | 31.50 | 107.00 | 191.00 |  | 143.90 | 16.80 | 108.00 | 165.00 |
|  | R | Probability occurrence of R horizon (%) | SoilGrids250m | 42.10 | 6.70 | 32.00 | 58.00 |  | 46.80 | 7.00 | 36.00 | 61.00 |
|  | SC | Soil organic carbon content (g kg^-1^) | SoilGrids250m | 102.30 | 20.00 | 61.00 | 130.00 |  | 32.90 | 5.50 | 24.00 | 53.00 |
|  | BD | Bulk density of the fine earth fraction (< 2mm) (kg m^-3^) | SoilGrids250m | 1092.70 | 59.90 | 1036.00 | 1218.00 |  | 1171.90 | 31.80 | 1039.00 | 1222.00 |
|  | CLAY | Percentage of clay in soil (Weight %) | SoilGrids250m | 38.40 | 1.00 | 37.00 | 40.00 |  | 37.70 | 4.60 | 34.00 | 47.00 |
|  | CF | Coarse fragments (Volumetric %) | SoilGrids250m | 24.00 | 1.60 | 21.00 | 25.00 |  | 21.30 | 0.80 | 20.00 | 24.00 |
|  | SAND | Percentage of sand in soil (Weight %) | SoilGrids250m | 32.90 | 0.70 | 32.00 | 34.00 |  | 31.60 | 3.40 | 25.00 | 35.00 |
|  | SILT | Percentage of silt in soil (Weight %) | SoilGrids250m | 27.40 | 1.00 | 26.00 | 29.00 |  | 30.80 | 1.30 | 28.00 | 33.00 |
|  | CEC | Cation-exchange capacity (cmol(c) kg^-1^) | SoilGrids250m | 29.00 | 2.40 | 27.00 | 36.00 |  | 28.70 | 1.20 | 27.00 | 32.00 |
|  | SS | Soil organic carbon stock (ton ha^-1^) | SoilGrids250m | 39.50 | 5.10 | 32.00 | 50.00 |  | 54.20 | 13.10 | 39.00 | 108.00 |
|  | pH_H_2_O | Soil pH in H_2_O solution | SoilGrids250m | 6.50 | 0.20 | 6.3.0 | 7.00 |  | 6.50 | 0.50 | 5.6.0 | 7.00 |
|  | pH_KCl | Soil pH in KCl solution | SoilGrids250m | 5.60 | 0.10 | 5.5.0 | 5.9.0 |  | 5.60 | 0.60 | 4.4.0 | 6.20 |
|  | FSC | Full soil code | INEGI | 12.40 | 4.70 | 6.00 | 18.00 |  | 18.30 | 3.10 | 14.00 | 24.00 |
| Geology | Geo | Geological units | INEGI | 8.00 | 0.00 | 8.00 | 8.00 |  | 12.90 | 8.60 | 5.00 | 27.00 |
|  | LIT | Lithostratigraphy | INEGI | 14.00 | 0.00 | 14.00 | 14.00 |  | 12.90 | 1.80 | 10.00 | 14.00 |
| Topo- | ASP | Aspect (º) | INEGI | 88.20 | 104.10 | 11.31 | 286.89 |  | 150.60 | 135.80 | 1.74 | 349.42 |
| graphy | CU | Curvature | INEGI | 0.02 | 0.24 | -0.31 | 0.39 |  | 0.21 | 0.33 | -0.22 | 0.86 |
|  | PLC | Plan curvature | INEGI | 0.03 | 0.12 | -0.19 | 0.23 |  | 0.02 | 0.09 | -0.11 | 0.19 |
|  | PRC | Profile curvature | INEGI | 0.01 | 0.15 | -0.25 | 0.16 |  | -0.18 | 0.28 | -0.80 | 0.11 |
|  | SLP | Slope (%) | INEGI | 16.76 | 10.03 | 9.10 | 33.79 |  | 24.69 | 10.70 | 6.53 | 48.21 |
|  | TSI | Terrain shape index | INEGI | -0.01 | 0.17 | -0.27 | 0.21 |  | -0.14 | 0.22 | -0.59 | 0.15 |
|  | WI | Wetness index | INEGI | 11.83 | 1.31 | 10.22 | 14.19 |  | 10.74 | 0.73 | 9.81 | 13.19 |
