# Supplementary material for: Assisted migration and the rare endemic plant species: the case of two endangered Mexican spruces
Source: PeerJ. 2022 Aug 3;10:e13812. doi: 10.7717/peerj.13812 (PMC9356587; doi:10.7717/peerj.13812)
Supplement: Supplemental Information 5 — The categories of probability of presences (p), the pixel count for each category of presence, and the real presences/absences of Picea martinezii and Picea mexicana on the corresponding predicted areas by the models are shown. [file peerj-10-13812-s005.docx]

| Species | *p* | Pixel count^[1]^ | Real presences (RP)^[2]^ | %RP | Real absences (RA)^[2]^ | %RA |
| --- | --- | --- | --- | --- | --- | --- |
| *Picea martinezii* | < 0.5 | - | 0 | 0.0 | All pixels | 100.0 |
|  | 0.5-0.6 | 12 | 0 | 0.0 | 12 | 100.0 |
|  | 0.6-0.7 | 3 | 1 | 33.3 | 2 | 66.7 |
|  | 0.7-0.8 | 0 | - | - | - | - |
|  | 0.8-0.9 | 1 | 1 | 100.0 | 0 | 0.0 |
|  | 0.9-1.0 | 6 | 6 | 100.0 | 0 | 0.0 |
|  |  |  |  |  |  |  |
| *Picea mexicana* | < 0.5 | - | 0 | 0.0 | All pixels | 100.0 |
|  | 0.5-0.6 | 16 | 0 | 0.0 | 16 | 100.0 |
|  | 0.6-0.7 | 15 | 2 | 13.3 | 13 | 86.7 |
|  | 0.7-0.8 | 14 | 2 | 14.3 | 12 | 85.7 |
|  | 0.8-0.9 | 9 | 5 | 55.6 | 4 | 44.4 |
|  | 0.9-1.0 | 10 | 9 | 90.0 | 1 | 10.0 |

^[1]^ Pixels of 30-arc s = 0.72 km^2^ (average of the pixel size at latitudes from 23º to 25º, where both species occur).

^[2]^ Real presences/absences show the number of pixels where the species are present or absent.
